# The Cognitive, Behavioral and Interpersonal Impacts of Virtual Practice with Short Health Videos on Chinese Ageing Women: A Discursive Approach

**DOI:** 10.3390/ijerph19127173

**Published:** 2022-06-11

**Authors:** Hongyan Liang, Huimin Pang

**Affiliations:** School of Journalism and Communication, Shanxi University, Taiyuan 030006, China; meggypang@sxu.edu.cn

**Keywords:** short health videos, Chinese ageing women, cognition, behavior, interpersonal, discourse analysis, transitivity analysis, generic structure

## Abstract

In the digital era, the health information presented on virtual platforms plays a pivotal role in supporting people’s active and healthy life. The ageing, especially ageing women, are more likely to seek and accept health information through online media platforms. The study shows that short health videos on social media platforms are extremely popular among ageing women in China for the accessing of virtual coaching. Adopting the qualitative methodology of in-depth interview and discourse analysis, the study investigates virtual coaching with short health video practice among 39 Chinese ageing women in different fields, who are all over sixty years old. Specifically, with the analytical tools of transitivity and generic structure analysis, the study explores the impacts of short health videos on Chinese ageing women’s cognition, behavior and interpersonal relationships. The result shows that virtual practice and coaching via short health videos can build health awareness and a dynamic new lifestyle, and motivate women to positively practice physical activity and maintain positive interpersonal relationships. Factors affecting the effectiveness of short health videos are discussed for future research in the field of modeling and intervention.

## 1. Introduction

As a convenient and effective means of conveying health and medical information to the public, short health videos on social media platforms have been popular among the ageing in China, penetrating into their daily life and behavioral interactions. Exposed to digital platforms, ageing women, who are reported as more relationship-oriented or more vulnerable in some specific health areas in the existing research [1,2,3], are more easily attracted to the diverse contents of short videos because they have a greater desire to obtain more health information, have more diverse emotional needs and a stronger willingness to get feedback. Therefore, the study attempts to observe whether video-based virtual health practice can promote ageing female users’ specific health cognition, behavior, and significant interpersonal improvement.

Regarding the topics of short health videos and ageing groups’ virtual health behavior, scholars at home and abroad have studied the topic from different perspectives.

### 1.1. Short Health Videos

The research on short health videos has been conducted from multiple perspectives. From the perspective of current and developmental situations, scholars [4,5] have found that the popularization of short health videos can be improved in content selection, users’ training and supervision mechanisms to avoid misunderstandings such as information distortion, unsuitable content and excessive entertainment. In terms of the representational characteristics, short videos are observed from the aspect of multimodal discourse and platform integration, media empowerment and symbolic interaction, narrative characteristics, and psychology and emotion, in order to eliminate untrue and inappropriate content and rumors during the communication process [6,7]. Regarding the communication channels, two main types of short videos are observed, official medical accounts, which seek new ways of health communication by exploring the operation of short video accounts in hospitals or institutions [8,9], and individual or team accounts, which release typical health videos and promote effective communication of health science content to the audience through short video platforms such as Tik Tok [10]. As to engagement with short videos, through frequent online searching and viewing and continuous accessing of relevant medical care activities, adults determine the correlation between health information and online medical consultation activities, and figure out the answers for health care providers, health facilities or medical treatment [11]. Concerning the virtual practice and coaching of short videos, the use of video has been demonstrated as an educational medium offering great potential advantages in modifying health behaviors, in a systematic review which examines video interventions across various medical specialties and diseases that seek to influence health behaviors, and assesses the effectiveness of videos relative to other media in changing health behaviors [12].

### 1.2. The Ageing Group’s Virtual Engagement with Health Information

With the penetration of information technology, the ageing are unconsciously involved in the digital wave. Especially, the sense of familiarity and presence created by short video platforms further strengthens their participation. This positive and convenient network interaction is beneficial not only to their individual health but also to their emotional connection with others. Specifically, the viewing and posting of short videos can create opportunities to alleviate social isolation and loneliness among the ageing. Especially when participating in a common activity with family members, friends and other network members, the ageing can meet their needs for information, interpersonal relationships, and social life, which reduces their loneliness and has a positive impact on their individual behavioral tendencies [13,14,15,16].

In terms of acceptance behaviors, a survey finds that the ageing are mainly affected by four factors when accepting information technology: needs satisfaction, perceived usability, support availability, and public acceptance, among which needs satisfaction and support availability are relatively more important [17]. Managing health is one of the important reasons why the ageing use information technology [18].

The social and emotional support from family members and friends, as well as the extension of provided network communication, further affects the social relations of the ageing group [19]. In terms of interpersonal interaction, the ageing have the intention of learning from each other and are willing to share experiences and health information with peers and their offspring, which can further promote their social participation and enhance their social adaptation [20,21]. An ageing community with diverse audio-based health communication information produced by the authoritative mainstream media can strengthen the ageing group’s information reception channels and capabilities [22,23].

### 1.3. The Present Research

In the existing literature, research on short video users mainly focuses on the youth group, and those on the ageing mainly concern the digital gap, intergenerational compensation, life improvement, etc., among which health videos are briefly overviewed as a tool element. In addition, current research on the health of ageing women is mainly concentrated on the fields of sports, medicine and others, focusing on the impact of physiological functions on their body, psychology and behavior, focusing on the specific topics of cancer, sex, suicide and depression [24,25]. In a word, there is little empirical investigation and systematic analysis of the cognitive, behavioral and interpersonal impacts of virtual practice and coaching from short videos for Chinese ageing women.

Therefore, based on the existing literature, the study, taking Chinese ageing women as an important group in the video-based information audience, investigates the impacts of virtual practice and coaching using short videos from the perspective of cognition, behavior and interpersonal relationships.

## 2. Materials and Methods

### 2.1. Participants and Inclusion Criteria

In order to gain a firsthand understanding of Chinese ageing women’s cognition, behavior and interpersonal relationship with virtual practice and coaching via short videos, 47 participants were recruited by 40 postgraduates at the School of Journalism and Communication, Shanxi University during the winter vacation of 2022. These postgraduates have professional knowledge and skills in qualitative methods in social studies and were informed of the research objectives before the interview. Most of the participants are the postgraduates’ family members or relatives or neighbors, who are mostly from the urban areas or the different counties of Shanxi Province in China. The inclusion criteria for selection were: (1) female over sixty years; and (2) possessing a smart phone and having had the experience of viewing the short health videos for at least half a year.

### 2.2. Methods and Procedures

The exploration of social media interaction can be approached by various methods, among which qualitative research has been widely and continuously chosen by researchers [26]. Two types of qualitative methods are adopted in this study: in-depth interview as the data collection methodology to collect the essential material for the survey, and discourse analysis as the data analytical methodology to uncover the interviewees’ experiences in their meaning-making during virtual practice and coaching via short videos.

#### 2.2.1. In-Depth Interview

In-depth interview is a widely employed and valued qualitative research methodology which involves comprehensive conversations between the researcher and interviewee, with a task-based exploration of the interviewee’s subjective experiences, meaning-making, accounting processes, and unspoken assumptions about life and the social world in general [27]. An in-depth interview is more complex than simply asking questions, because the researcher must understand the interviewee’s perceptions, opinions, and lived experience. In this study, it is adopted as a suitable method for collecting data from Chinese ageing women to observe their cognitive, behavioral and interpersonal changes by engaging with short videos on popular social media platforms.

In-depth interviews provide access to the context of people’s behavior, opening a way for the researcher to understand the meaning of that behavior. A basic assumption in in-depth interviewing research is that the meaning people make of their experience affects the way they live that experience [28].

The information from the interviewees is very useful for understanding the current situation regarding Chinese ageing women’s engagement with short health videos. The questions specifically asked during the interviews are: (1) What are the reasons for watching short videos? (2) Do you think short videos are beneficial? Has there been any change in your health awareness? (3) Have you made any adjustment to your living or eating habits after watching the short videos? (4) Will you follow the content or advice of the short videos? If not, what factors affect this? (5) Would you like to share or recommend the short videos with/to your family members or friends? Who do you usually share with? By forwarding the links or by recounting the information? (6) Has watching short videos influenced or improved your family life? Has there been any change or improvement in building your family relationships?

#### 2.2.2. Discourse Analysis

Discourse analysis is the discipline devoted to the investigation of the relationship between form and function in verbal communication [29], in which scholars maintain that discourse and society are so deeply intertwined that discourse can rightly be considered as a social process and practice [30]. Language is a way people construct their own realities and experiences, their identities, and the relationships between themselves. This study assumes that the interviewees’ virtual engagement with the short videos will be encoded and evident in the language or discourse they use.

Regarding the theoretical and methodological framework for analyzing the interviewees’ narratives, the study takes a discourse-oriented perspective based on Systemic Functional Linguistics [31,32] and Critical Discourse Analysis [33,34]. Specifically, two analytical tools are adopted in this study: transitivity analysis and generic structure analysis.

The central idea in Systemic Functional Linguistics is that language is a semiotic system of choice making. Transitivity analysis carries out the description of the representation of social events and social actors and construes the experiential meaning through clauses that are constituted of participants, processes and circumstances as is displayed in Table 1, which offers a way to decode the specific information regarding how the participants experience the world and their construction of social reality. The process types include: material process, behavioral process, mental process, verbal process, relational process and existential process, and each of them “provides its own model or schema construing a particular domain of experience as a figure of a particular kind” [32] (p. 170). In this sense, transitivity analysis could be usefully employed to grasp the actions, thoughts and feelings of the interviewees.

Genre is defined as “a socially ratified way of using language in connection with a particular type of social activity” [34] (p. 14). Genre analysis is primarily concerned with staging by observing the internal relations of texts, including analysis of semantic, grammatical, vocabulary and phonological relations [33]. The “beginning-middle-ending” structure formed by sequences of stages realizes discourse as a social practice [35] (p. 194). The generic structure analysis in this study can fully observe how the interviewees relate to their different stages of experiences in their meaning-making.

Starting by examining the transitivity patterns and generic structures of the interviewees’ utterances based on the discourse analytic process, an interpretation and explanation of the cognitive, behavioral and interpersonal impacts of the virtual practice and coaching of short health videos on Chinese ageing women will be categorized and summarized. The detailed procedures for the study are displayed in Table 2.

## 3. Results

### 3.1. General Characteristics of Participants and Practice with Short Health Videos

During the data collection stage, the data of eight participants were excluded due to missing crucial information for the analysis. Thus, the final sample comprises 39 Chinese women, whose age ranges from 60 to 80 years (Mean = 64.5). The basic information on the participants is listed in Table 3.

Educational level and job experience. The participants have varied educational degrees: 18 of them have high school education, six have a college degree, 10 have middle school educational level and five only have the experience of primary education. Most of them have job experience, as is shown in Table 3.

Health situation. Table 3 also displays the participants’ general health situation. 20 of them report that they are in good health and 19 state that they have some disease or previous experience of symptoms, such as hypertension and diabetes.

Living conditions. Most of the participants live with family, except that No. 15 lives alone and No. 37 lives with three room mates in a dormitory of a hotel where she works as a cook.

Practice with short health videos. In general, the participants have an intensive and varied use of smart phones, including viewing short videos for at least between half a year to five or six years. The daily frequency for practicing with short videos is relatively high, from half an hour to three or four hours a day. The social media platforms the participants usually rely on include Kwai, Tik Tok, WeChat Short Video, Toutiao, etc., among which Kwai and Tik Tok are much more popular. At the beginning of their practice with social media platforms, most of them report that their children or grandchildren offered coaching or help with downloading the APPs. The contents of the short videos they mainly focus on include senile diseases, such as high blood pressure and diabetes, aerobics or dancing, and healthy eating. The providers of the videos include two rough categories: official medical institutions, and individuals and relevant groups. The former is mainly composed of health agencies and hospital accounts, and the latter includes both professional and non-professional health practitioners.

### 3.2. Cognitive Change Construed by Gradable Process and Sequential Structure

The analysis of the sample to assess the cognitive change of the interviewees shows that 32 give the definitely positive answer that virtual practice and coaching via short videos are beneficial, and their cognition regarding health problems has changed after watching the videos. One interviewee (No. 12), a doctor in a village, gives the negative answer that there is no change in her cognition. The rest of the interviewees respond positively but in a partial degree or hesitation; for example, No. 27 answers “duōshǎo yǒu yìdiǎn (*a little bit*)”, No. 39 responds “méiyǒu duōdàde biànhuà BA (*not much has been changed BA*)”.

As is exemplified in Table 4, during the interview part of the cognitive changes, the most frequent processes produced by the interviewees are mental processes, relational processes and behavioral processes. These representative process types display their own cognitive states and change processes.

The cognitive mental process “juéde” (*think*) or “rènwéi” (*think*) and the attributive relational process “shì” (*be*) are the most recurring and important meaning resources that enable the interviewees to depict their cognitive state. The circumstances are mainly the positive comments on the effect of watching short videos, such as “tǐnghǎo” (*very good*), “yǒu dàolǐ” (*reasonable*), ”yǒuyì”, “yǒuhǎochù”, “shòuyìfěiqiǎn” (*beneficial*), “yǒubāngzhù” (*helpful*), “yǒusuǒgǎibiàn” (*changed*). More specifically, the interviewees construe their cognitive change by using the mental process of “zhùyì” (*pay attention to*), “guānzhù” (*pay attention to*), “zhǎngwòle” (*acquire*), “zhīdào” (*know*), “bāngzhù wǒ liǎojiě le” (*help me understand*). The behavioral processes, such as “cǎinà” (*adopt*) and “fǎngzhào” (*imitate*) are graded ones in the interviewees’ cognitive change, i.e., they begin to accept useful information and take actions after virtual coaching on the short video health platforms.

The interviewees’ cognitive processes are not only construed by the gradable process types, but also by their arrangement of the generic structure. As is exemplified in Table 4, three main types of generic structures are used: the contrast between their cognition before and after their viewing of the short health video with optional examples, as in the narration of No. 1, No. 5 and No. 18; the sequence of modification stages between their cognitive changes and the actions to be taken, as in the narration of No. 4; and the affirmation that the short videos are definitely beneficial along with some optional comments, as is indicated by No. 21 and No. 30.

### 3.3. Behavioral Change Represented by Action Description and Contrastive Pattern

In terms of the behavioral impacts, the interviewees’ living habits and exercises following the virtual coaching are investigated. 34 interviewees, accounting for 87.2%, believe that there are changes in their living habits, two do not think they have experienced change, and the rest do not think they have seen much change. As to the question whether they can follow the virtual coaching in order to exercise, 32, accounting for 82.1%, give the affirmative answer, three do not think they have experienced much change, and four admit that they do not change at all.

The changes in living habits narrated by the ageing women are displayed as different aspects regarding their health, such as time rescheduling, as in No. 14′s narration “wǎnshàng yào zǎoshuì, bùyào chāoguò shíyīdiǎn” (*go to bed early at night, no more than eleven o’clock*), eating habits and other aspects in the interviewees’ narration. In this part of the statement, as is best exemplified in the following two transcriptions of No. 20 and No. 30, the main type of generic structure is exemplification, in which the contrasts between “yǐqián” or “zhīqián” (*before*) and “xiànzài” (*now*) or after watching a short video are made.

1. **Yǐqián** BA, búài chūqù, ài zàijiālǐ dāizhe. **Xiànzài**le **kànle yǐhòu**, rènwéi rénjiā shuōde hái tǐngduì de, yǒuxiē fánxīnshì, chūqù hé biérén duō jiāoliú jiāoliú, duō wánwan, duō duànliàn duànliàn, huíjiāhòu zhège fánxīnshì jiù wàngle, tǐnghǎode. **Xiànzài** wǒ měitiān zǎoshàng qǐlái shǒuxiān hē yībēi wēnkāishuǐ, ránhòu chuāndàihǎo jiù hé línjūmen qīdiǎnláizhōng qù cūnwài mànzǒu. Wǒ **yǐqián** chīfàn bǐjiào kǒuwèizhòng, **xiànzài** bùgǎn kǒuwèi zhòngle, gào jiālǐderén yěshì, chīfàn yào shǎochīyán, kǒuwèi chīde qīng dàn yīdiǎn, píngshí zài yǐnshí shàng shǎochī yóunìde. Píngshí chīxìliáng, chīxiē shànshí xiānwéi, búyào lǎochī báimiàn, dàmǐ; duōchī qiáomàimiàn, yùmǐmiàn, fǎnzhèngshì duōchī cūliáng BA. (No. 20)
  ***Before****, I didn’t like going out, I liked staying at home. **After watching it**, I think what they say is quite right. When there are some annoying things, go out to communicate more with others, play more exercises, I will forget the annoying things after returning home, which is good. **Now** after I wake up every morning, first I will drink a glass of warm water, then get dressed and go out for a slow walk outside the village at seven o’clock with my neighbors. I **used to** have a heavy taste in eating, but **now** I don’t dare to have a heavy taste. I also tell my family that we should eat less salt and less greasy food. Usually we eat refined grains, we should eat some dietary fiber, such as more buckwheat noodles, cornmeal, anyway, eat more coarse grains, instead of white noodles or rice all the time.*
2. Bǐrú zài shēnghuó xíguàn fāngmiàn, shuìjiào zhīqián yòng rèshuǐ pàojiǎo, yībān huì pào bàngèduō xiǎoshí, zuòyizuò shǒubù yǐjí tóubù de ànmó duànliàn, yǒuzhùyú bāngzhù shuìmián; zài yǐnshí xíguàn fāngmiàn, **zhīqián** kǒuwèi piānxián, chǎocài zuò fàn shíyán huì fàngdeduō, **xiànzài** yǐjīng shǎochī shíyán, háiyǒu xīnlà cìjì de shíwù, yóunì de ròulèi chīde yěshǎole, chīde shūcài, húluóbo, dòuzhìpǐn huì duōyīxiē. (No. 30)
  *For example, in terms of living habits, I wash my feet in hot water before going to bed, usually for more than half an hour, and do a massage on hands and head to help sleep; in terms of eating habits, **before** I prefer the salty taste and like putting more salt in cooking. **Now** I eat less salty, spicy food and less greasy meat, and eat more vegetables, carrots and soy products.*

By observing the sample, as to the question whether they can follow the virtual coaching in order to exercise, the study finds that most of the interviewees can do so, as is indicated in Table 5, for example, “kànshàng zhíbò, zuòzuò pāidǎcāo, zuòzuò ànmó cāo” (*watching the live video to do some slapping and some massage exercises,* No. 11), “gēnzhe shìpín zuò jǐngzhuīcāo” (*following the video to do cervical spine exercises,* No. 33). The reasons for virtual coaching are mainly because they have diseases (as is expressed by No. 11, 25, 33) or they are not strong (as No. 39). Most of them can follow virtual coaching to exercise for a long term (*two or three years, many years*). In the interviewees’ narration, the effects of exercise through virtual coaching are obvious, such as “yǒu xiàoguǒ” (*effective*)*,* “tǐng shíjì” (*very practical*)*,* “tǐng guǎnyòng” (*very helpful*)*,* “tǐng yǒu jīngshén” (*very energetic*)*,* “hǎole” (*improved*)*,* “qíngkuàng hǎoduōle” (*much better*)*,* ”huǎnjiě” (*relieved*). Expectations are optional in their statement, but generally they expect that virtual coaching will be effective and they can be healthier.

As for the interviewees who don’t follow virtual coaching; the reasons can be attributed to their relatively young age and strong and healthy body, for example, No. 15, who is 63, states “Ànlǐ yīnggāi gǎibiàn, dànshì zìjǐ kòngzhì bùzhù, gǎibiàn bùliǎo, kànlái háishì niánlíng xiǎo deguò” (*I should make a change, but I can’t control myself. I can’t change it. It seems that I am still too young*); No. 16, who is 72, says “Lǎnde zuò, yuányīn jiùshì shēntǐ háikěyǐ, yàoshì jīngcháng shēngbìng, kěndìng jiù gèng zhùzhòng yīxiē” (*I am too lazy to do it. The reason is that I am still healthy. If I get sick often, I must pay more attention*).

### 3.4. Interpersonal Improvement Realized by Sharing Process

As to the question whether they would share short videos with others, among the 39 interviewees, 36 give an affirmative answer. In terms of the way of sharing or recommendation, 11 (31%) share by forwarding the link, 8 (22%) use oral narration, 16 (44%) use both of the above, and 32 interviewees believe that viewing or following these videos has a positive impact on improving family life.

The observation of transitivity finds the processes adopted by participants are mainly material processes, that is, the action of sharing, or verbal processes, the action of telling. The analysis of process types, Goal or Receiver (the one to which/whom the process is extended), and adverbial elements can best display the interviewees’ sharing activities.

36 of the interviewees would like to share useful videos to others. As to the processes, besides unilaterally recommending, sharing or forwarding the information, 53% would take part in more communication, for example, “**jiǎnjié** yìdiǎn jiùshì, yǒuyòng de, zhòngyào de, **fēnxiǎng** gěi qítā rén” (*extract a little bit useful and important information and share it with others,* No. 1), “gēn biérén **shìpín, tàntǎo** duǎn shìpín lǐ de yǎngshēng zhīshi” (*video-chatting with others, discussing the health knowledge in the short videos*, No. 4), “gěi tāmen **jiǎngshù** yíbiàn, huòzhě gěi tāmen **shìfàn** yíxià zhège dòngzuò” (*retell the information to them, or show them how to do it,* No. 11), tōngcháng huì **zhuǎnfā** dào qúnlǐ, tóngshí yěhuì **shuōjǐjù** (*usually retweet to the group and also add a few words*, No. 33).

The Goals or Receivers are not limited to their family members or relatives. The interviewees would share or communicate with a wide range of people, for example, “fāgěi **tóngxuéqún**, **chūzhōng tóngxué, shīfàn tóngxué, tóngshì**” (*forward it to all the groups, including the groups of relatives and friends, junior middle school classmates, normal college classmates, and colleagues,* No. 4), “yígè wéichíle sìnián de dàyuē, 50 rén de **fěnsīqún**” (*a fan group with about 50 members that has been maintained for four years*, No. 33), “yīkuài dǎpái de **péngyǒumen**” (*the friends with whom I play poker,* No. 38). Among the Goals or Receivers, the participants also target their information to certain groups, for example, “yóuqí shì bú shàngbān de rén” (*especially for those who are not working,* No. 4),” “Wǒ **mèimei** ma, tā búshì tángniàobìng…. Duì tā yǒuyì, wǒ jiù gěi tā fāguòqù le.” (*My younger sister is a diabetic patient.... If it is good for her, I’ll send it to her.* No. 14).

The adverbs of degree or the mood particles used by the interviewees can clearly display their attitudes towards interpersonal behavior. Referring to their sharing activities, in the following transcriptions, the expressions “**dōu**yào fāchūqù ne” “**Yóuqí**” used by No. 4 (a teacher) and “**kěài** zhuǎnfā YA” “**tèbié** ài fā”, “**měitiān** dōuyào fā, **méiyǒu y**ì**tiān bù fā de**” used by No. 10 (a technical worker) indicate their great desire and willingness to share the videos which they think useful and helpful to their family members, relatives and friends.

3. Fāzài péngyǒuquān le hé qīnyǒumen de qúnlǐ, háiyǒu huì fāgěi tóngxuéqún, chūzhōng tóngxué, shīfàn tóngxué, tóngshì, **dōuyào fāchūqù NE**. **Yóuqí** shì bú shàngbān de rén wǒ jiù gěi **duō** fāxiē liànjiē, yěràng tāmen zàijiā biān kānháizi biān duànliàn shēntǐ. (No. 4)
  *I send (the health information) to the moments (on Wechat) and all the groups, including the groups of relatives and friends, junior middle school classmates, normal college classmates, and colleagues. Especially for those who are not working, I send more links, and let them exercise at home while taking care of their grandchildren.*
4. … wǒ yuánlái yěshì, **ài fābù YA**. Jiùshì nà shíhòu gānggāng názhe shǒujī wán MA HA, AIYA **kě xiǎng zhuǎnfā NE**, hǎoxiàng biérén dōu kàn bùjiàn, wǒ gǎnjǐn bǎ zhège zhuǎnfā chūqù HA…. hǎoxiàng yuánlái fǎnzhèng **tèbié ài fā**, **měitiān dōuyào fā**, **méiyǒu y**ì**tiān bù fā de**, **yǒushíhòu hái fā hǎojǐtiáo NE**. En **fēnxiǎng dào péngyǒu quān HA, fāchūqù**. … jiùshì gāng náshàng shǒujī wánde shíhòu **kě xiǎng fā NE**. **AIYA, kě yào fā NE, fā gěi zhège fā gěi nàge**. (No. 10)
  *… I liked forwarding so much, and loved posting. When I was first playing with my mobile phone, I loved forwarding so much. It seemed that no one else could see it, so I quickly forwarded. …It seemed that I really loved posting, every day. Sometimes I posted several pieces in one day. Well, I shared them on moments, sending all of them out. …When I first got my phone, I just wanted to post. I wanted to forward links, to this one or to that one.*

The analysis of the sample also shows that sharing short health videos improve the interviewees’ family life, and 32 of the interviewees give an affirmation and positive comments, for example, “Dàjiā dōu juéde tǐng kāixīn de.” (*Everyone feels very happy.* No. 23), “Mànman péngyǒu yě biànduōle” (*Gradually I have more friends.* No. 38), “Gēn tāde jiāoliú duōle yǐhòu, xiǎoxífuer zhīdàole wǒde yòngyì, duì wǒ yěyǒule gǎiguān.” (*After communicating with her more, the little daughter-in-law understands my intentions and has a changed attitude towards me.* No. 39).

Two (No. 17 and No. 31, both are workers) use the expressions “shāowēi yǒuxiē” and “shāowēi gǎishànle yìdiǎn BA” to indicate very few or slight improvements in their family relationship. Four indicate that the sharing of the videos doesn’t have any or much impact on family life or relationship: “méiyǒu shénme biànhuà” (No. 22, a housewife), “biànhuà búdà” (No. 26, a teacher), “duì jiātíng shēnghuó yǐngxiǎng búdà BA” (No. 33, a housewife), and “méiyǒu tàidàde yǐngxiǎng” (No. 35, a housewife). However, No. 26 adds that the change mainly lies in diet (yǐnshí fāngmiàn huì yǒu yǐngxiǎng) and No. 33 admits that the change is mainly reflected in the individual (zhǔyào shì duì gèrén de yǐngxiǎng).

While reporting the positive impacts, six of the interviewees (15%) refer to negative impacts, for example, “yǒu shíhòu kàn shìpín kàn shàngyǐn, kànde shíjiān tài cháng, yǐngxiǎngle jiārén de xiūxí” (*Sometimes I get addicted to watching videos and spend a long time, which affects my family members.* No. 21), “gēn jiārén de jiāoliú biàn shǎole, xiánxiá shíjiān jīběn huāzàile kànshìpín shàng” (*Communication with family members has been decreased, and most of the leisure time has been spent on watching videos.* No. 36).

## 4. Discussion

The study qualitatively explores ageing women’s perceptions and behavioral and interpersonal experiences of short health videos on popular social media platforms in China. The processes of these modifications are best displayed in the interviewees’ narration, discursively construed by the representational realization of process types and generic structures. At the same time, some variables affecting the interviewees’ understanding, adopting and sharing of the health information are also implied and identified, such as previous experience of symptoms, interactive communication, spatial distance, and the need for and motivation towards health information. The variables provide insight for further research into ageing people’s engagement with short health videos.

### 4.1. Meaning-Making of Modifications Construed in the Process Types and Generic Structures

Since language reflects and constitutes social structures, discourse analysis, centralizing language as a guiding element to understand the functioning of social dynamics, offers the means to discuss social phenomena through language not only from the perspective of knowledge production, but also from that of society in general. Language is the means by which people construct their own realities and experiences, their identities, and the relationships between themselves [32,36]. Through language, people position themselves and express their opinions, evaluations, emotions, tastes, and values [37].

The study investigates from a discursive perspective the narratives produced by 39 Chinese ageing women, focusing on their cognitive, behavioral and interpersonal changes after the virtual practice and coaching derived from short health videos. By analyzing the interviewees’ narratives, the study displays the ageing women’s perspectives about current discourses on this modification process, as well as their viewpoints on virtual engagement with these short videos, and investigates how modifications are construed in their narratives.

In general, the modification process from cognition to behavior and then to interpersonal improvement is a gradable one.

At the individual level, according to the knowledge, attitude and practice (KAP) theory [38,39], people establish positive and correct beliefs and attitudes based on their understanding of health knowledge and information, and then take the initiative to form healthy behaviors or changes. The transition from cognition to behavior requires learning and accepting knowledge first, then forming a belief in health promotion, and finally producing behaviors that promote health or eliminating behaviors that endanger health. The interviewees’ cognitive, behavioral and interpersonal processes, are not only construed by gradable process types, but also by their arrangement of the generic structure. By unveiling the interviewees’ representations of the most frequent process types, the study finds that the cognitive and behavioral change processes are construed from the cognitive mental process, “juéde” (*think*) or “rènwéi” (*think*) and the attributive relational process “shì” (*be*) with positive comments, such as ”yǒuyì” (*beneficial*), “yǒubāngzhù” (*helpful*) about their cognitive change after adopting the virtual health information by expressing “zhǎngwòle” (*acquire*), “bāngzhù wǒ liǎojiě le” (*help me understand*), and even graded behavioral processes, such as “cǎinà” (*adopt*), “fǎngzhào” (*imitate*) or “gēnzhe shìpín zuò...” (*follow the video to do...*). The main generic arrangements, i.e., the contrast between their cognition before and after their watching the short videos with optional examples, the sequence of cognitive changes and actions to be taken, and the affirmation that these videos are definitely beneficial, along with some optional comments, uncover the representation of modification by contrasting, sequence and affirmation patterns.

At the interpersonal level, in the Chinese context of collectivist culture, the ageing need more information technology especially regarding health information to connect with others, due to their closer interpersonal relationships [40]. They may have a higher tendency to employ behaviors that maintain relationships with others [41]. Affected by cognitive and behavioral changes, the interviewees consciously or unconsciously show their influences on the persons around them. As to whether they would like to share the videos by forwarding or retelling, the processes adopted by the interviewees are mainly material processes, the action of sharing, such as “zhuǎnfā” (*forward*), or verbal processes, the action of telling, such as “tàntǎo” (*discuss),* “jiǎngshù” (*retell*), shuōjǐjù (*add a few words*). The Goals or Receivers with whom the participants share or communicate are also not limited to their family members or relatives, but to a wide range of people, especially to those who need the specific health information. The adverbs of degree or the mood particles used by the participants vividly indicate their desire and willingness to share the short videos with their family members, relatives and friends. As to whether sharing short health videos improves the interviewees’ family life, most of them display an affirmative attitude by unveiling their representations with positive comments and evaluations.

### 4.2. Factors Affecting Cognitive, Behavioral and Interpersonal Modification

As is indicated in many studies [17,42], perceived usefulness is widely recognized to be the most important variable in predicting information technology acceptance. Through analysis of the interviews, it is found that perceived usefulness is the most important factor affecting the interviewees’ cognitive and behavioral modifications and are more specifically embodied in two aspects: their health situation or previous experience of symptoms, and age.

The analysis in Section 3 indicates that the health situation, that is whether they have some disease or previous experience of symptoms, is an important factor affecting ageing women’s cognitive and behavioral changes. The analysis of the interviews uncovers that ageing women with some diseases, such as hypertension and diabetes, are more likely to experience changes in their understanding after exposure to related short videos, believe that their health understanding has changed, and hope that their health will be improved day by day. For example, 71-year-old No. 7, suffering from high blood pressure and high blood lipids, talks about the changes in her diet after watching the videos: “*I now cook with less oil and less salt, which is good for blood vessels... get more sun exposure, because some experts say that calcium in food is just a part of it and cannot be fully absorbed. It is good to go out and get some sun exposure.”* In contrast, for those without any disease, their willingness and motivation towards behavioral change are not very strong. For example, No. 16, who is 72 years old, says that she is still healthy and too lazy, but she would pay more attention if she often got sick. Therefore, ageing women with diseases are more inclined to accept health information in short videos, and make adjustments to their lifestyle habits and eating habits or physical exercises.

According to the interviews, there is also a strong correlation between age and changes in cognition and behavior. Some ageing women in their early 60s make no major changes in acquired behaviors after watching health videos; on the contrary, elder women are more willing to receive virtual health coaching and adjust their health behaviors after viewing the short videos. For example, the 61-year-old No. 19 responds that there has not much change in her cognition and exercise behavior; and the 63-year-old No. 15 also mentions in the interview that she can’t control herself and make changes because she thinks she is still young.

Interpersonal communication plays an important role in changing attitudes and plays a key role in persuading individuals to adopt healthy behaviors [43,44]. At the interpersonal level, the analysis also indicates the main factors affecting the interviewees’ changes, namely the communicator’s specific need for the health information, interactive communication, and spatial distance.

The interpersonal relationship structure is determined by the needs of human beings [45]. Some interviewees in the study would like to share short videos with a particular communicator, with specific contents relating to his/her special physical condition, for example, No. 14, a teacher, focuses on sharing relevant health knowledge with her younger sister because her sister has diabetes; No. 28 stresses the importance of health to her husband because “*my husband has high blood lipids. At the beginning, I imitated the short videos to make some light meals... Gradually, he began to accept it. And now my husband often comes home for dinner, and the family relationship is more harmonious”;* and No. 30 often shares health information on eating habits to family members because “*the granddaughter has bad eating habits, likes fried foods such as hamburgers, and rarely eats vegetables, which leads to her weak physical condition and often being ill*”. Besides family members and relatives, the interviewees also target their information to a certain group, who have a great need for specific health information, for example, those who are not working (No. 4) and those who are in their eighties (No. 11).

In addition, the study also finds that interactive communication between the interviewee and the communicator has an important impact on the willingness to share. According to Altman & Taylor [46], communication between people is a process starting from a simple chat, through to showing their true feelings as intimate friends. What is more, interactive communication is important in interpersonal improvement. If the participant gets a response from the communicator, it signals that they can continue to communicate. If there is no response and it is only a unilateral communication, the participants’ willingness to share may be reduced in the future. Some participants in the interviews, such as No. 4, No. 7 and No. 32, are refuted by their children when sharing short health videos due to their differences in intergenerational attitudes, thereby reducing their willingness to share, as is narrated by No. 4 “*Children are young and they have their own opinions. The views and living habits between the ageing and young are also different. My sharing health knowledge with them will be opposed,... so later on I will not communicate with them about this. No longer share!”.*

Although the rapid development of transportation and information makes co-presence face-to-face interaction between people increasingly unnecessary [45], to the ageing women participating in this study spatial distance is still one of the indispensable environmental determinants of their willingness to share, for example, No. 6, a farmer, would not share health information with her family members because they don’t live together. Interviewees, such as No. 17, a retired worker, No. 22, a housewife, No. 23, a retired middle school Chinese teacher, and No. 32, a housewife, tend to share short videos with family members or friends because they live at a relatively close distance.

Besides the above mentioned essential factors, others such as occupation and technology skill have less impact on interpersonal improvement, but are also mentioned by the interviewees. For example, No. 13, a retired doctor, says “*Maybe it has something to do with my previous job. If I think it’s good, I want to promote it to everyone so that they can enjoy it”;* No. 6 accounts the partial reason why she doesn’t like sharing health information, “*I don’t know how to forward it, I just watch it myself*”.

## 5. Conclusions

The study provides clear evidence that short health videos on social media platforms can build ageing women’s health awareness, motivate behavioral modification and improve interpersonal relationships. Factors affecting these changes can be taken into consideration for future research in the field of short health videos in terms of modeling and intervention. At the same time, the study also attempts to apply and extend the notion of transitivity analysis and generic structure analysis to health communication research. It shows the great potential of discourse analysis, as a complementary investigation tool in analyzing qualitative material, especially on the effectiveness of short health videos in modifying cognition, behavior and interpersonal relationship.

## Figures and Tables

**Table 1:** Elements in transitivity analysis and their realization.

Participant	Process Type	Circumstance
nominal group	verbal group	adverbial group or prepositional phrase

**Table 2:** Procedures for the study.

Stage	Procedure
Data collection stage	Contacting the interviewees formally and getting their consents; Conducting the interviews individually and recording them; Transcribing the interviews; Observing and discussing the transcription; Excluding the invalid ones.
Data analytical stage	Conducting the transitivity analysis; Summarizing the generic structures; Interpreting and explaining the analytical results; Discussion on the analysis.

**Table 3:** Basic information on the participants.

No.	Name	Age	Job	Disease
1	LYR	61	ancient architecture surveyor	/
2	LCX	61	unemployed	/
3	WLY	65	accountant	/
4	WJF	67	teacher	/
5	WYH	65	civil servant	/
6	LCX	72	farmer	hypertension, diabetes
7	HCL	71	worker	hypertension, hyperlipidemia
8	ZEF	64	teacher	/
9	ZSC	60	accountant	/
10	AJP	64	retiree	/
11	HXY	80	file clerk	hypertension, coronary heart disease, osteoarthritis
12	LJP	60	village doctor	/
13	RS	61	doctor	/
14	LYX	79	teacher	joint problems
15	SPX	63	school administrator	/
16	LRL	72	teacher	/
17	LMY	60	retiree	/
18	YLX	61	retiree	/
19	ZHY	61	worker	hypertension
20	WXP	63	housewife	diabetes, hypertension
21	ZXL	70	custodian of the bureau	bronchitis, dry eye
22	JYX	72	unemployed	hypertension
23	NXM	67	teacher	/
24	SYF	62	retiree	/
25	WXZ	66	farmer	hypertension, leg pain
26	ZBS	62	teacher	hypertension
27	CXY	60	cleaner	hypertension, hyperlipidemia
28	WXY	60	canteen staff	hypertension
29	LXH	65	retiree	osteoarthritis
30	DQM	60	self-employed	hypertension, insomnia
31	DGY	60	worker	/
32	ZGR	62	housewife	/
33	RHF	60	unemployed	/
34	BCX	62	unemployed	hypertension
35	SJX	60	housewife	/
36	GML	62	worker	hyperglycemia
37	YFJ	62	cook	diabetes
38	LGL	73	housewife	hypertension
39	WXH	62	housewife	rheumatism

**Table 4:** Analysis of the interviewees’ narration about cognitive change.

No.	Narration	Main Process Type	Generic Structure
1	Yǐqián bú tài **zhùyì**, tōngguò kàn zhège duǎn shìpín zìjǐ yě **yǒusuǒgǎibiàn.** *I didn’t pay much attention to it before, but I have changed myself by watching short video.*	mental process relational process	Contrast (before + now)
4	**Juéde** tǐnghǎo yǒuyì, wǒ jiù **cǎiyòng**le, zài rìcháng shēnghuó xíguàn zhōng, gāi **zhùyì** nǎxiē, yùndòng duànliàn shēntǐ, yǐnshí shàng yǒuyì de wǒ dōuhuì **cǎinà.** *I think it is good and helpful, so I adopt it. In my daily life, I adopt all the information about what I should pay attention to, physical exercise, and useful living and diet habits.*	mental process behavioral process	Sequence (cognition + action)
5	Tīngle zhīhòu shì **shòuyìfěiqiǎn** de. Zài jiànkāng rènshí shàng yǒule **hěndà de biànhuà**, cóng yuánláide bùdǒng, tōngguò jiǎngjiě, biàn **zhǎngwòle** hěnduō de bǎohù shēntǐ de zhīshi, **zhīdào** zěnyàng bǎohù shēntǐ, zěnyàng duànliàn shēntǐ, nǎge shíjiān yīnggāi shài tàiyang A, zuò zǎocāo. Bìngqiě **xuédàole** hěnduō de yǐnshí zhùyì shìxiàng, **zhīdàole** zěnyàng qù yǎngshēng zhè yī fāngmiàn de zhīshi, cóng bùdǒng dào jìnyíbù de **liǎojiě**, **jiāshēnle** duì jiànkāng zhīshi de **rènshi**. *Listening to it is very beneficial. There has been a great change in my health awareness. By watching the coaching, I have decreased the original ignorance, mastered a lot of knowledge about body promotion, knowing how to protect, how to exercise, when to bask in the sun, and do morning exercises; and learned a lot of dietary precautions, getting knowledge about how to maintain health, from not knowing to further understanding, deepening the understanding of health knowledge.*	relational process mental process	contrast (before + now) + examples
18	Bǐrúshuō wǒ gāngkāishǐ jiǎoténg, zhǐshì **rènwéi** shì quēgài, jiù kāishǐ bǔgài, hòumiàn tōngguò kàn duǎnshìpín, **zhīdào** shì qítā shēntǐ bùwèi yě kěyǐ yǐngxiǎng dào jiǎohòugēn téng, tuǐténg zhè yílèi de. *For example, at the beginning of my feet pain, I just thought it was calcium deficiency, so I started supplementing calcium. Later, by watching short videos, I learn that other body problems can also cause heel pain and leg pain.*	mental process behavioral process	example + contrast (before + now)
21	**Rènwéi** yǒuyì, yǒu biànhuà, **gèngjiā guānzhù** zìshēn jiànkāng zhuàngkuàng, **gèngjiā zhùzhòng** yǎngshēng. *Think it is beneficial. There are changes, paying more attention to my own health, and paying more attention to health promotion.*	mental process relational process	affirmation
30	Kěndìng shì yǒu hǎochù de, **bāngzhù** wǒ **liǎojiě** le bùshǎo jiànkāng fāngmiàn de zhīshi, duì rìcháng de jíbìng yùfáng zhìliáo yǒu hěnhǎo de bāngzhù, jùyǒu hěn qiáng de cānkǎojiàzhí. *It is definitely beneficial. It helps* *me to understand a lot of health knowledge, which is very helpful for daily disease prevention and treatment, and has a strong reference value.*	relational process mental process	affirmation + comment

**Table 5:** Interviewees’ generic statement about the behavioral change through virtual coaching.

No.	Action	Reason	Duration	Effect	Expectation
6	chūqù duànliàn, gēbó shuǎi shuǎi, rénjiā jiāode gǎndào shǒumále zěnme huódòng, yǒushíhòu xīn yǒudiǎn huāngle zuò shénme dòngzuò *go out to exercise, toss arms, the videos teach me how to move when my hands are numb, and what to do when I feel a little panicked*	/	jiānchíle yīliǎng nián le *have been insisting for a year or two*	yǒu xiàoguǒ, yǒuxiē yùndòng háishì tǐng shíjì, tǐng guǎnyòng de *effective, and some exercises are quite practical and effective*	*/*
11	kàn shàng zhíbò, zuòzuò pāidǎcāo, zuòzuò ànmó cāo *watch the live video, do some slapping, do some massage exercise*	tuǐ bùhǎo *my legs are not good*	měitiān bàn xiǎoshí *half an hour every day*	/	/
23	xuédiǎner jiànshēn cāo, hé dàjiā yìqǐ tiàoyitiào, duōliànliàn, méishìerle duō zǒuzǒulù *learn some aerobics, dance with others, practice more, go for more walks*	/	qǐmǎ bàngè xiǎoshí, jiānchíle hǎoduōnián le *at least half an hour, have been insisting for many years*	bāngzhù hěn dà, juéde měitiān tǐng yǒu jīngshén de, gǎnjué dào gànge jiāwùhuóerle, huòzhě gànge shénme shìer yě bútàilèi *be great helpful, feel very energetic every day, not too tired when doing housework or any other thing*	*/*
25	gēnzhe zuòyizuò cāo, měitiān zǎoshàng yěhuì chūqù duànliàn *follow to do some* *exercises,* *go out to exercise* *every morning*	jiānbǎng téng, gēbó téng *my shoulders and arms* *ache*	jiānchíle sān nián *insist on for* *three years*	jǐngzhuī yě hǎole, gēbó yěnéng táiqǐláile *my cervical spine is improved, and my arms can* *also* *be lifted*	qīdài shǎo débìng, shēntǐ hǎoyīdiǎn, bù máfan érnǚmen *looking forward to less sickness,* *being* *healthier, and no trouble for the children*
33	gēnzhe shìpín zuò jǐngzhuīcāo *follow the video to do cervical spine exercises*	jǐngzhuī bù hǎo, jǐngzhuībìng fēicháng yánzhòng, shènzhì gēbó dōu tái bùqǐlái *the cervical spine was not good, cervical spondylosis was very serious, and even couldn’t lift the arms*	jiānchíle liǎngsān niánle *have been doing it for two or three years*	xiànzài qíngkuàng hǎoduōle *things are much better now*	xīwàng jìxù gēnzhe duànliàn, shēntǐ yuèlái yuèhǎo *hope to continue to exercise and get better and better*
39	mófǎng jiù xiàng guǎngchǎngwǔ yīyàng de jiànshēncāo, zàijiā mófǎng zhe zuò *imitate the aerobics like square dancing, imitate it at home*	shēntǐ búshì hěn zhuàngshí *not very strong*	jiānchíle yǒuyī nián ba dàgài *insist for about a year*	kěyǐ huǎnjiě *can be relieved* zhège shìpín háishì bù yīyàng de *(watching) the videos is quite different*	qīdài jiùshì zhèxiē yǒu xiàoguǒ *expect it will be effective*

## Data Availability

Data and materials are available on request from the corresponding author.
